# Supplementary material for: Natural Flavonol, Myricetin, Enhances the Function and Survival of Cryopreserved Hepatocytes In Vitro and In Vivo
Source: Int J Mol Sci. 2019 Dec 4;20(24):6123. doi: 10.3390/ijms20246123 (PMC6940939; doi:10.3390/ijms20246123)
Supplement: Supplementary file 1 [file ijms-20-06123-s001.pdf]

Table S1. Q-PCR primer sequence and product size.

Gene Product	Primer Sequence		Amplicon Size (bp)
	Forward	Reverse	
Albumin	TTTATGCCCCGGAACTCCTTT	AGTCTCTGTTTGGCAGACGAA	148
CYP3A4	AAGTCGCCTCGAAGATACACA	AAGGAGAGAACACTGCTCGTG	174
Alpha1-antitrypsin	CCTATAACGTCACCGACCTCG	TGGAAGCATTCGTGGATCTTG	208
Tyrosine aminotransferase	CTGGACTCGGGCAAATATAATGG	GTCCTTAGCTTCTAGGGGTGC	111
Tryptophan 2, 3-dioxygenase	TCCTCAGGCTATCACTACCTGC	ATCTTCGGTATCCAGTGTCGG	110
Hepatocyte nuclear factor-4 alpha	CGAAGGTCAAGCTATGAGGACA	ATCTGCGATGCTGGCAATCT	141
Cytokeratin 18	GGCATCCAGAACGAGAAGGAG	ATTGTCCACAGTATTTGCGAAGA	216
Carbamoylphosphate synthetase I	TTTAGGGCAATGGCTACAGG	GTTCTGCAAGAGCTGGGTTC	373
Ornithine transcarbamylase	CTGATTACCTCACGCTCC	TCTCCTCTTCTCGTCCCA	308
GAPDH [1]	TGCACCACCAACTGCTTAGC	GGCATGGACTGTGGTCATGAG	87

[1] Glyceraldehyde-3-phosphate dehydrogenase.

Table S2. Primer design and location in genotyping of ornithine transcarbamylase (OTCD) and Severe combined immunodeficiency (SCID) mouse.

Target Gene	Primer Sequence	
	Forward	Reverse
OTCD [1]	ATGTGAGTGATGCTTCTCCTGGGT	TCGATTGAGATAGCTGGTGCAAGTA
Prkdc [2]	GAGAAAAGGAGGATCATGGATTCAAGAAATAAATGTAACG	CCTAAGAGTCACTTTCTCCATTTACACAGTGAAGTGCC
MF	TGGTATCCACAACATAAAATACGCTAA	
WR		TGGCCCCTGCTAACTTTCTCTTAGCA

[1] Primer amplicon includes exon 4 which has a mutation of CAC ([117] histidine) to GAC (aspartate). After the enzyme digestion (5′ CACNN ∣ NNGTG 3′), gel electrophoresis shows a 658 base pair (bp) band (diseased), a 558 bp band (wild type), or 658 and 558 bp bands (heterozygous). Sequence around exon 4: 5′-
TTCTCGATTGAGATAGCTGGTGCAAGTACTGATGCCTCATAATTTGGTTAACATTTAGTTCTTTCGTTTTCCCCTCTCAATACATTCACTGTCTTTTTCTTT
TGTTCTAGGCTTTGCTCTGCTGGGAGGACACCCTTCCTTTCTTACCACACAAGACATTGACTTGGGTGTGAATGAAAGTCTCACAGACACCGCTCGGTTTG
TAAAACTTTTCTTCCTTCCAAAGTTTATTTCAAACTCTGATGGGTTAGTTTAAAAGAGAAGATGATGCTTCTCCTTAGATAATGGTCTCCCCCTTTCGGTGT
CTTTTGTACCTTCTCCCCTTTACAGGGCTCTTTATTTAATGTTTGAAACGCCTCTCTTTCATTCTGTAGCCCCTTTTTAGTGTCTTACTTTGTTCCCATTGTTAT
GATAAAGGCCAAGGTAAAAGCAATTTGGGCAGGAAAGGGTTATTTCATCTTAAAGTCCACATAAATCCATTTTAAGATGAAATAAACCTTTTCCTAACC
AAAGAAAGAAAGCCAGGGCAAGAACTCAAGACAAAAACCAGGATGCAGGAACTCAAGCAGAGGCCATAGAAGAATGGTGTTTAATGGCTTGCTCAAA
CTGCCTTCTTAAACCATCCAGGACCACCCACCCAGGAGAAGCATCACTCACATTGAGTGGGGCCCTCTTCTATTGATTGGCAATCAAG -3′. Yellow area indicates exon 4 which includes a mutation of CAC ([117] histidine) to GAC (aspartate) (double underline). Primer pairs were indicated by under lines. [2] According to the polymerase chain reaction using confronting two-pair primers [34]. Gel electrophoresis shows 257 and 180 bp bands (homozygous SCID), 257, 180, and 101 bp bands (heterozygous SCID), and 257 and 101 bp bands (wild type).

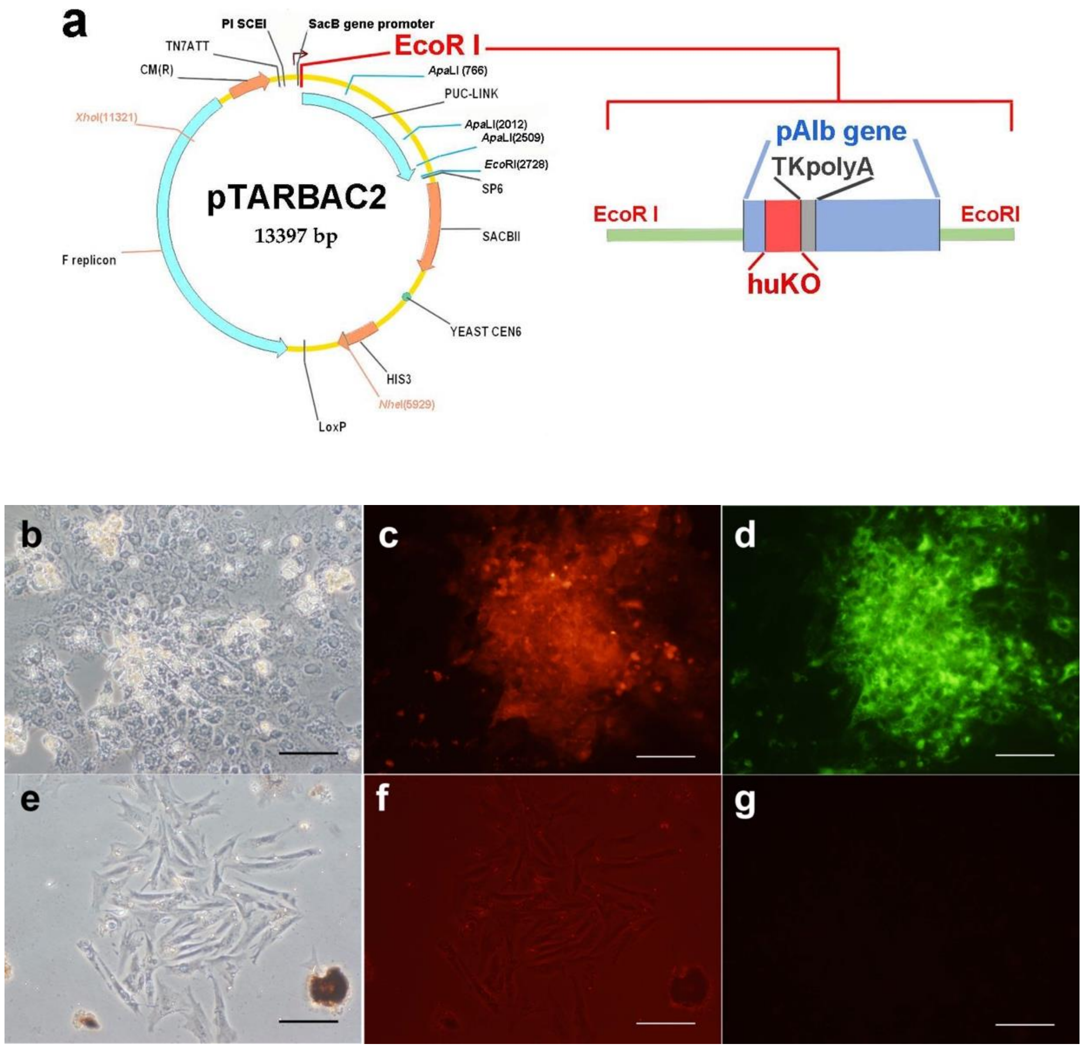

Supplementary Figure 1. (**a**) Construction of transgene used for the establishment of kusabira orange transgenic pig. pAlb gene; promotor of procine albumin. huKO; humanized kusabira orange gene. Kusabira gene originally from Fungia concinna was modified to enhance the expression efficacy by replacing the Fungia concinna-specific codons with mammalian ones [19,21]. TKpolyA; HSV-thymidine kinase (TK) promoter. (**b–g**) Cultured hepatocytes isolated from the pig. (**b–d**)

Colony of parenchymal hepatocytes at culture day 2. (**e–g**) Colony of non-parenchymal hepatocytes at culture day 3. (**b**,**e**) Phase contrast micrographs. (**c**,**f**) Fluorescent micrographs of kusabira orange. (**d**,**g**) Fluorescent micrographs of albumin staining (FITC). Scale bar: 200 μm.
